# A realist evaluation approach to explaining the role of context in the impact of a complex eHealth intervention for improving prevention of cardiovascular disease

**DOI:** 10.1186/s12913-020-05597-5

**Published:** 2020-08-18

**Authors:** Genevieve Coorey, David Peiris, Lis Neubeck, Julie Redfern

**Affiliations:** 1 Faculty of Medicine and Health, School of Public Health, The University of Sydney, Sydney, New South Wales Australia; 2 The George Institute for Global Health, Sydney, New South Wales Australia; 3 Faculty of Medicine, The University of New South Wales, Sydney, New South Wales Australia; 4 School of Health and Social Care, Edinburgh Napier University, Edinburgh, UK; 5 Faculty of Medicine and Health, Westmead Applied Research Centre, The University of Sydney, Sydney, New South Wales Australia

**Keywords:** eHealth, Electronic health record, Cardiovascular disease, Realist evaluation, Prevention, Complex intervention, Context, Mechanism

## Abstract

**Background:**

Reduction of cardiovascular disease (CVD) is a worldwide health priority and innovative uses of technology-based interventions may assist patients with improving prevention behaviours. Targeting these interventions to recipients most likely to benefit requires understanding how contexts of use influence responsiveness to the intervention, and how this interaction favours or discourages health behaviour. Using a realist evaluation approach, the aim of this study was to examine the contextual factors influencing behaviour change within a multi-feature eHealth intervention with personalised data integration from the primary care electronic health record (EHR).

**Methods:**

Realist evaluation of qualitative data from the Consumer Navigation of Electronic Cardiovascular Tools (CONNECT) randomised trial (*N* = 934). Thirty-six participants from the intervention group (*N* = 486) who had completed 12 months of study follow-up were interviewed. Coding of transcripts was structured around configurations of contexts, mechanisms, and outcomes of intervention use. Contextual narratives were derived from thematic analysis of the interviews.

**Results:**

Mechanisms favouring positive health behaviour occurred when participants responded to four interactive features of the intervention. Facilitating mechanisms included greater cognitive engagement whereby participants perceived value and benefit, and felt motivated, confident and incentivised. Participants moved from being unconcerned (or unaware) to more task-oriented engagement with personal CVD risk profile and prevention. Increased personalisation occurred when modifiable CVD risk factors became relatable to lifestyle behaviour; and experiences of feeling greater agency/self-efficacy emerged. Use and non-use of the intervention were influenced by four overarching narratives within the individual’s micro-level and meso-level environments: illness experiences; receptiveness to risk and prevention information; history of the doctor-patient relationship; and relationship with technology.

**Conclusions:**

Intervention-context interactions are central to understanding how change mechanisms activate within complex interventions to exert their impact on recipients. Intervention use and non-use were context-dependent, underscoring the need for further research to target eHealth innovations to those most likely to benefit.

## Contributions to the literature

- Mechanisms by which complex eHealth interventions lead to outcomes are hard to observe and depend on the various contexts in which they are used. There is limited research about how a complex web-based application that is integrated with the electronic health record helps patients to improve health-related behaviours.
- Utilising a theory-driven evaluation approach, we elucidated the cognitive and emotional mechanisms at play in how the intervention worked in people with, or at increased risk of, cardiovascular disease.
- Characterising the important contextual influences on patients’ engagement with eHealth interventions contributes to understanding use and non-use of technology to support disease prevention in varied service settings and populations.

## Background

### Rationale for process evaluation of complex eHealth interventions

Complex interventions are defined by their multiple components; or multiple target user groups; the variability in their expected outcomes; and the multiple and/or difficult behaviours required by program recipients or providers [1]. Furthermore, uniformity and identical conditions of use do not hold for interventions introduced into social systems that are constantly changing, regardless of the presence of the intervention [2]. For a complex intervention introduced into social environments, a traditional experimental evaluation that attempts to estimate aggregate effectiveness tends to neglect the influence of contextual and intervention factors in the outcomes produced [3]. Described as black box evaluation, [4] outcomes-focussed evaluations may overlook underlying socio-cultural influences of importance to future implementation [5]. This has been raised as a criticism of intervention research in the area of eHealth, [6] in which internet-related technologies attempt to “support, enable, promote and enhance health and augment the efficacy and efficiency of the process of healthcare” [7]. (page 2) By contrast, a process-focussed evaluation aims to explain the pathways by which the intervention effects occurred (the *mechanisms*) and their interaction with context [8]. Further, a theory-driven approach to conceptualising and interpreting the process evaluation is useful because it offers a logic by which to explore causal mechanisms in the relationship between program inputs, mediating contextual factors and program outputs [3].

### Realist evaluation as a theory-driven approach to conceptualising an evaluation

Realist evaluation is an explanation-driven form of enquiry underpinned by critical realism, which holds that causation stems from generative mechanisms that activate when an intervention is introduced into a system [2]. Realist evaluation assumes that programs are complex interventions introduced into social systems, which themselves are complex [9]. Interventions offer the recipients resources, to which the recipients do or do not respond, depending on context. The premise is that an intervention *per se* is not what works; rather, its recipients make it work depending on how they respond to the resources it offers them [9]. This process of receiving, interpreting and acting is the *mechanism* and the mechanism generates the change(s) in, for example, behaviour or attitude [3]. Mechanisms tend not to be observable; what is observable are their effects [2]. Thus, realist evaluation elucidates contextual influences and meanings that help explain what it is about an intervention that makes it work. Furthermore, derivation of context-mechanism-outcome (C-M-O) configurations are proposed to generate testable theories for future research about why recipients within certain contexts respond to one or more aspects of an intervention [10].

#### Intervention

In the current study, we focus on evaluation of a multi-feature interactive eHealth intervention with integration of electronic health record (EHR) data that was designed for self-directed home-based use by consumers. The intervention had the overall intent of improving the recipient’s cardiovascular disease (CVD) risk factor profile by facilitating health-related behaviour change, including increased engagement with care providers. Development and design of the web application has been extensively detailed previously [11]. Briefly, the key personalised features were absolute CVD risk score estimation; updateable biometric and blood test data with self-monitoring option; current prescription medications with consumer information; lifestyle goal setting and tracking for physical activity, healthier eating, smoking cessation; and mental well-being. Information resources and a social chat forum were included. When not logged in, participants could opt into receiving by text message and/or email semi-personalised health messages and lifestyle tips derived from national CVD prevention guidelines. The many contexts in which the intervention would be used were unknown/unknowable at the time of implementation.

#### Program theory

Theories about how and in what circumstances an intervention is proposed to influence behaviour are known in realist evaluation as program theories [12]. Fogg’s model of behaviour change, which focuses on the convergence of motivation, capacity and trigger, [13] and persuasive software design principles, [14] were two key theoretical foundations (the program theories) underpinning the intended impact of the intervention. Persuasive principles, including goal tracking, self-monitoring, praise, reminders and system content from a trustworthy source, were intended to strengthen the Fogg triad assumptions. The logic of the intervention was further depicted diagrammatically in a process model of stages from intervention inputs to outputs [15].

### Framing contextual environments as hierarchies of influence on behaviour

Social context influences on intervention uptake or health-related behaviour are often focussed on specific illnesses, [16–18] cultural groups, [19–21] and organisation-level innovations [5]. In terms of how health behaviours arise and change, frameworks that advocate integration of behavioural and social sciences tend to emphasise the dominance of social structure over individual characteristics. For example, a depiction of society-behaviour-biology linkages across the lifespan, nested in complex relationships between social and physical environments, framed the linkages in a hierarchy [22]. This framework designates micro-, meso-, macro-, and global-levels of structures or environments in the scope of public health interventions, and emphasises that dynamics between the levels above the individual help explain human behaviour. Other research, however, has shown how adaptations of the hierarchy can be a useful way to frame the socio-cultural narratives that individuals use to explain their behaviour [23]. Taking a hierarchical view of contexts, interventions in areas as diverse as welfare services, [24] diabetes self-management, [25, 26] cardiac rehabilitation, [27] and emergency department workflow [28] have been examined using realist evaluation to understand how mechanisms were activated. Examples are limited for EHR-integrated eHealth interventions in the broad setting of primary and secondary CVD prevention. However, future development and implementation of such interventions would benefit from greater understanding of influential contextual narratives on recipient responsiveness. Therefore, the aim of this study was to use a realist evaluation approach to elucidate contextual factors at play in participant responses to the eHealth intervention and describe mechanisms by which the impact on outcomes arose.

## Methods

### Design and setting

Qualitative study in which methods were informed by realist evaluation principles. Rather than selecting a study sample that is representative of a broad population, evaluation with a realist perspective requires observing or exploring causation in purposively selected cases in order to build the theory about what worked, for whom, how, and in what circumstances [29]. Each selected case sits in an essentially limitless open social system. A case is chosen because it produces a configuration of a setting (context) with the resources of the intervention and the ideas, intentions, choices and abilities within the recipient (mechanisms) leading to a behaviour change (outcome) [30].

The study forms part of the process evaluation of the Consumer Navigation of Electronic Cardiovascular Tools (CONNECT) RCT, [ANZCTR ID:12613000715774] [31]. In a randomised controlled trial (*n* = 934), the eHealth intervention was tested for effectiveness in improving CVD risk factor profile, including the proportion of days covered by guideline-recommended medications, over a 12-month follow-up period. The comparator group received usual health care without access to the intervention. Adults with, or at increased risk of, CVD who had access to an internet-enabled device at least once per month and who could provide written informed consent were eligible to participate in the RCT. Recruitment was from 24 primary health care services in Sydney, Australia. Eligible patients at each health service were identified using the clinical software system. Patients were invited to take part via a postal letter from their health service or general practice. Those who then enrolled were randomly allocated to a treatment arm. Of those who were invited (*n* = 3552), 26% (*n* = 934) enrolled. Of these, 52% (*n* = 486) were allocated to the intervention group and 48% (*n* = 448) to the control group. Trial results are pending. Ethical approval was obtained from the University of Sydney (Reference 2013/716) and the New South Wales Aboriginal Health and Medical Research Council (Reference 959/13).

### Recruitment and data collection

The process of contacting study participants to take part in interviews, confirming interview arrangements, obtaining written informed consent, and conducting the audio-recorded interviews is detailed elsewhere [15] using qualitative reporting criteria (see Supplementary File 1). Briefly, 53 participants who had completed 12 months of study follow up were approached to take part in a one-hour interview and 36 (68%) agreed to do so. Each interview was conducted in a private room at the health care service from where the participant had been recruited. A semi-structured interview guide was used.

Consistent with the realist approach, a purposive sampling strategy was used. Purposive sampling is a strategy in which particular settings, persons or activities are selected deliberately because they are able to provide information that is relevant to the research question or goals [29]. Variation within the interviewees’ experiences of the intervention and personal factors was sought in order to identify the range of potential C-M-O configurations at play. Therefore, sampling categories included level of education, CVD risk status, baseline eHealth literacy, pace of uptake of new technology, and login frequency (including non-use of the intervention). Inclusion of counterfactual cases is suggested to buffer threats to validity that could arise from perceived uniformity of self-reported accounts of the intervention [29]. Intervention use was determined from routinely-collected web log data about login activity and screen use, and variable self-reported technology use and eHealth literacy score at study baseline.

In line with the realist approach to interviewing cases, the researcher used knowledge of the original program theories and intended outcomes to elicit causal and circumstantial triggers from the many subjective intervention experiences [10]. Therefore, the hypothesised impact of the various intervention features was tested in the interviews and formed the core of the interview guide. The researcher conveyed to the interviewee the underlying concepts and prompted interviewees to appraise their experience of the intervention against the proposed constructs (Table 1). Follow-up questions were asked if responses were yes/no. Responses such as feelings, beliefs, fears, values, intentions, and so on, were sought to illuminate how interaction with the intervention in individual situations affected reasoning and decision-making in respect of health behaviour [29]. In this way, influences on how the intervention worked other than those proposed in the original program theories were expected to emerge from a range of participant accounts and offer greater explanatory depth to how the intervention worked [32].

**Table 1:** Examples of interview questions aimed at confirming or falsifying intervention assumptions

**Looking for mechanisms or unintended responses:**	
*▪ “Your CONNECT website was linked in with your health record at the general practice. We expected that this could be an important innovation that would interest people, and that it might prompt greater interest in their own health situation. Was that the case for you?”* [questioned further if answer was yes/no]	
*▪ “When we built the heart risk screen, we assumed that people would react to it by thinking about their personal heart health risk and possible steps they could take to improve it, maybe to discuss it a little more with their GP. Did that heart risk screen have an impact for you?”*	
*▪ “As with many other technology-based programs for improving health, we included screens where you could set and track personalised goals because we suspected that this is a feature people like to use to see their achievements and keep motivated. It could be different for others. What do you think about it?*	
**Looking for mechanisms; exploring contexts of use:**	
*▪ “In what ways do you think technology can be helpful for people who are trying to increase healthy lifestyle behaviour? What features do you feel are important?”*	
*▪ “People are motivated to improve their heart health for many different reasons. For you, what were reasons to start or increase healthier behaviour?”*	
*▪ “Can you tell me about things in the CONNECT program that you read or used that helped you think about risk for heart disease, or made you want to make some lifestyle changes?”*	

Abbreviations: *CONNECT*, Consumer Navigation of Electronic Cardiovascular Tools; *GP* general practitioner

### Data analysis

To develop the C-M-O configurations, one researcher reviewed the interview transcripts for self-reported cognitive, attitudinal or behavioural changes in respect of cardiovascular health (O-outcomes). Each intervention resource that the participant described as helpful or influential (or not) was coded to a descriptor of their reasoning or decision-making, creating resource-response dyads (M-mechanisms), and selected quotes illustrated the responses. Each transcript was also coded to descriptors of the participant’s context (C) of program use. These descriptors generated the sub-themes of contexts of use. Two researchers then grouped the intervention-context interactions into overarching contextual narratives that illuminated why participants responded as they did. This step extended understanding of ‘what worked?’ for impact within this intervention to uncover the conditions that favoured or deterred its use. NVivo 12 Pro (QSR International Pty Ltd., Victoria, Australia) was used to facilitate organisation of the data.

## Results

### Participants

Thirty-six participants from the intervention arm of the RCT (*n* = 486) were interviewed. Their mean age was 67 years; 50% were male, and 50% had an existing diagnosis of CVD. Half of the interviewees had school-only education and most were retired (Table 2).

**Table 2:** Baseline characteristics of interview participants

	Interviewees (***n*** = 36)	RCT Cohort (n = 934)
**Age, mean (SD) years**	67 (8)	67.6 (8.1)
**Male % (n)**	50 (18)	76.7 (716)
**Highest completed educational qualification % (n)**		
School only	50 (18)	28.1 (262/931)
Undergraduate degree	16.7 (6)	19.7 (183/931)
Postgraduate degree or diploma	16.7 (6)	27.5 (256/931)
Technical/vocational qualification	16.7 (6)	24.7 (230/931)
**Employment status % (n)**		
Working	27.8 (10)	37.5 (335/894)
Retired	72.2 (26)	62.5 (559/894)
**CVD status % (n)**		
Existing CVD	50 (18)	41 (383)
High risk of CVD	50 (18)	59 (551)
**eHEALS**		
Total score ≥ 26% (n)	72.2 (26)	65.8 (613/931)
Total score < 26% (n)	27.8 (10)	34.2 (318/931)
Score, mean (SD)	27.7 (7.2)	27.0 (6.4)
**Self-reported uptake of new technology products % (n)**		
I am generally the first, or among the first	19.4 (7)	22.8 (213/933)
I am generally in the middle	50 (18)	49.4 (461/933)
I am generally the last, or among the last	30.6 (11)	27.8 (259/933)
**Login frequency**^a^ **% (n)**		
High users	61 (22)	40.4 (182/451)
Low users	28 (10)	46.8 (211/451)
Non-users	11 (4)	12.8 (58/451)

Abbreviations: *CVD* cardiovascular disease; *eHEALS* electronic health literacy scale; *SD* standard deviation

^a^High use: logged into the application at least once, in more than 3 months of follow-up period; Low use: logged in at least once, in 3 months or less of follow-up period; Non-use: logged in only once in total during follow-up period

Notes

1. Denominators are included where the denominator differed from the column total

2. Login frequency applies only to the intervention group (n = 486); denominator shown (*n* = 451) excludes those with no logged use of the intervention

Two discreet but related perspectives on the interview data results were derived from the transcripts. First, the change mechanisms described by participants in relation to specific features of the eHealth intervention; second, the overarching contextual narratives that influenced intervention use. The first perspective showed more granular insights into individual health-related behaviour. The second perspective was a broader view of important contextual factors that were derived from thematic analysis.

### Change mechanisms underlying the intervention features

Interviewees responses to four specific intervention features were positive or negative depending on contexts of use: (a) the EHR-derived risk score estimation and biometric and pathology risk profile; (b) CVD guideline-recommended lifestyle information and medication tips; (c) updateable medication list from the EHR, with consumer drug information; and (d) personalised goal setting and tracking with virtual rewards. The underlying mechanisms by which individual responses to these four features produced the reported outcomes were grouped by feature (see Supplementary File 2). Examples of change mechanisms that were triggered included feeling motivated, confident, and incentivised; and of moving from low/no concern (or low awareness) to concrete, task-oriented engagement with personal CVD risk profile and prevention management. In addition, participants experienced raised consciousness about the relationship of modifiable CVD risk factors with lifestyle behaviour, and greater agency/self-esteem in health behaviour. Action and outcomes followed from these changes. For each of the four listed intervention features, a case example illustrated how the response of the recipient triggered change when the intervention resources interacted with a context of use.

**Table:** 

**Case example (a): Receptiveness to personalised CVD risk profile information** **Resource:** Biometric data updated within the application A female interviewee, age range 50–60 yrs. described an outcome as: “if I’m waiting around at the chemist for my medicine to be dispensed, I’ll do my blood pressure and…see how I’m tracking.” **Circumstances (context):** “It [intervention] kind of coincided with...working closer to home in the past year, so it was an opportunity I guess to really do something. ...It was “right, I’ve got more time. I’m going to get healthier”. **Response to the resource:** “I actually did find that part of it interesting, whenever my doctor had made a change and you get an email and you think, oh what’s he done? I think that’s really valuable. It’s good to have that information.” Outside the healthcare encounter, she made connections between EHR-derived information presented in the application and her blood pressure control and took up blood pressure self-monitoring opportunities (the change mechanism that has activated in this context).

**Table:** 

**Case example (b): Receptiveness to prescriptive health information aimed at ‘nudging’ the recipient towards healthier lifestyle behaviour/choices** **Resource:** CVD guideline-recommended lifestyle information and medication tips. A male interviewee, age range 70–80 yrs. described an outcome as: “The study helped me apply some of the ideas that I’ve had.” **Circumstances (context):** “I need to get rid of some of this weight for a number of reasons, my heart and my bowel cancer, and I also have sleep apnoea. So if I could get some weight off, it’s going to help every one of those issues.” **Response to the resource was to perceive credible guidance from the intervention content because it concurred with advice from trusted providers:** “It goes in with what [GP] wants, what CONNECT is telling me and advising me and what the dietitian is telling me. It jolted me into doing the things that I should have been doing, like way back in 2013”. Trust stemmed from the alignment between information from several sources and encouraged the change from delay to action (the change mechanism that has activated in this context).

**Table:** 

**Case example (c): Prescription medication knowledge** **Resource**: Updateable medication list from the EHR, with consumer drug information A female interviewee, age range 50–60 yrs. described an outcome as: “I can ask questions when I go to the doctor…it empowered me to actually speak up and ask about things. That’s very different to what I normally do. I just go, do as I’m told, and I feel like I’m the one who needs to be told off.” **Circumstances (context):** “I would come to the doctor and I don’t ask. I just take a script and walk away and go and fill it. I was not looking after myself with my diabetes. I was taking the medication, and if it ran out, I wasn’t caring about it until some things came up. I started to lose my vision, my feet were burning and my blood pressure had gone up. So, the alarm bells were going off, like,” you’re in trouble” so I knew I had to do something. So, when an opportunity to go on the study, I thought, ‘Yeah, this might encourage me’.” **Response to the resource:** “I’m opening my records up, and looking at what my medication was and…there was information there and I just didn’t know that sort of stuff. But this was in your own house and you’re online and nobody is going to say, “God, you’re stupid,” because you just click a little button and the information comes up and you go, “Oh, so I’m taking that for that reason.” I’d do a bit of research and a bit of personal reading and I understand why it’s important. Whereas before…if I missed [medications] then I didn’t know and I didn’t care.” The resource was permissive for her adopting medication knowledge-seeking, which increased her confidence to participate more actively in health care encounters (the change mechanism that has activated in this context).

**Table:** 

**Case example (d): Tracking lifestyle health behaviour goals** **Resource:** Personalised goal setting and tracking with virtual rewards A male interviewee, age range 60–70 yrs. described an outcome as: “So all of a sudden, I’ll do the walk and I’ll do another one today, and then another one the next day.” **Circumstances (context):** “Blood pressure and heart disease, there seems to be a history of that through the family. After I left work I thought, “Oh yeah, I’ll be all right. I’ll surf every day and walk every day,” but it never really eventuated. After a while unless you’ve got something really planned... you’ll read the paper, and you tend to sit down, you become a little bit sedentary” **Response to the resource:** “I guess this came along and...became an opportunity to perhaps assess myself or keep an eye on how I’m doing things to see whether it has any impact. What do I want to do today, or what do I want to do this week? So at least it kept you focussed. Increased cognisance that his current lifestyle has implications for him avoiding illnesses of his parents; anticipation of benefit from the resource increased his motivation and control (the change mechanism that has activated in this context).	

### Key overall narratives of the intervention-context interaction

**Fig. 1:**
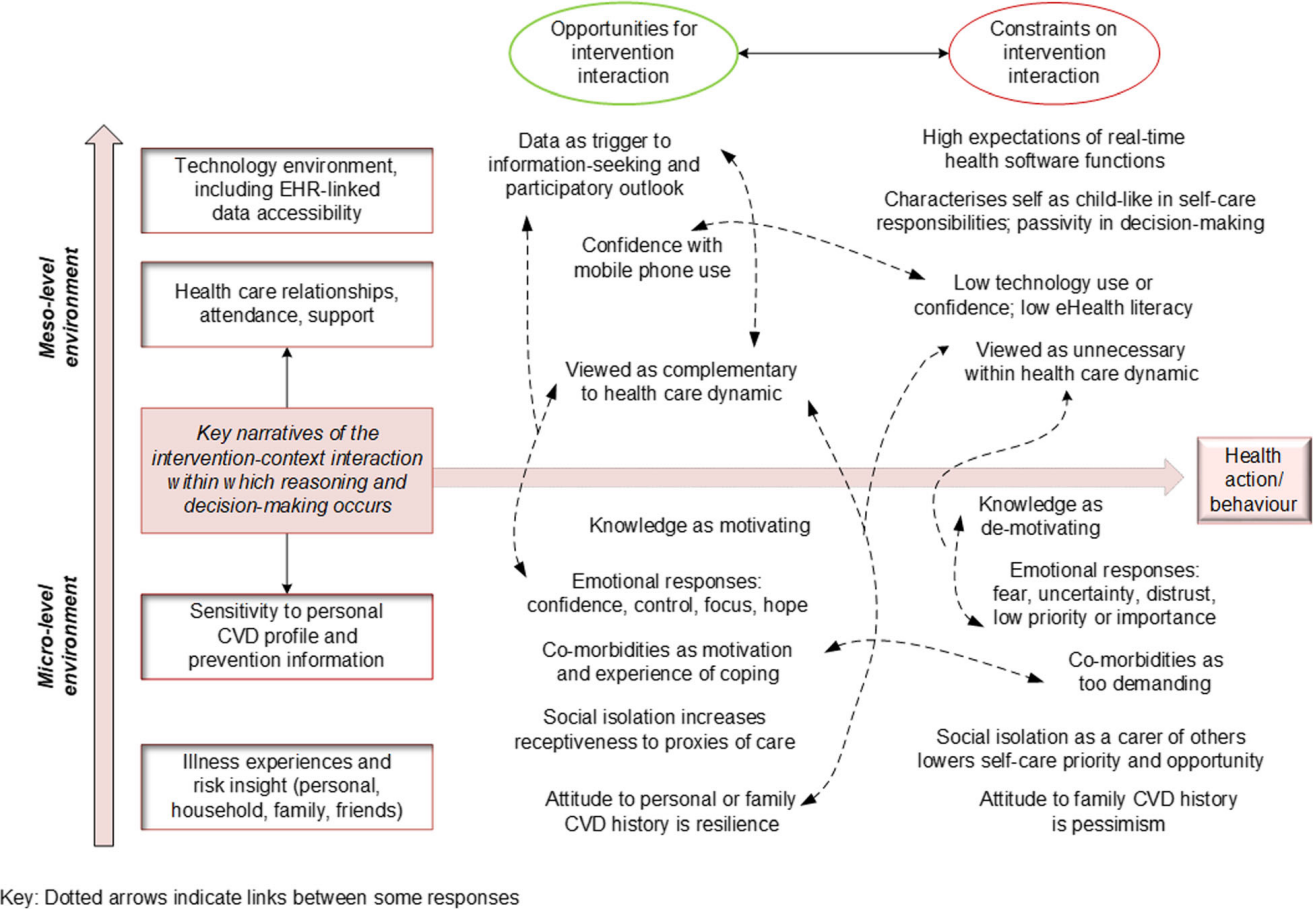
Contextual narratives within micro- and meso-level environments influencing responses to an eHealth intervention. Abbreviations: CVD, cardiovascular disease; EHR, electronic health record

Taking another perspective on the interview data, important contextual factors were identified from thematic analysis of the interviews. The contextual sub-themes around use and non-use of the intervention are described within Supplementary File 2, where they have been incorporated into detailed appraisal of C-M-O configurations. From the sub-themes, four overarching narratives within the micro- and meso-level environments of the patients’ lives emerged as influential on use of the intervention (Fig. 1).

#### Illness experience

Illness was a theme portrayed three ways by participants who described responsibility *to self* (to improve their health-related behaviour and avoid illness) and *of self* (the belief that doing so depended on their own efforts). First, personal illness (diagnosed CVD and/or other conditions, such as cancer or diabetes) and/or risk factors (for example overweight; hypertension) were described both as a barrier (attention needed for health problems today overshadows future disease prevention) and a driver of engagement (fear of health consequences but time enough left to make a difference to future prognosis for CVD). Several participants mentioned contextual changes that focussed them more actively on lifestyle behaviour that would help them live healthier and longer with their current conditions, for example, the arrival of grandchildren, and work-related factors such as retirement or re-location. Others described feeling wary of poor future health as they observed their parents’ health in decline. The unexpected intensification of drug therapy to control CVD risk factors influenced intervention engagement. Changed reasoning was expressed in various ways:

*“I treat it all as a challenge, to an extent. [The target values] is where I should be…Yeah, it gets into a mindset; I’ve got to get there, somehow, so…if it wasn’t visible to me, I’d just go along with the flow.“ (*Male, age range 60-70 yrs.)

*“I've eaten too much. I've drunk too much. I've smoked all my life. I think it's time to say, well, let’s see if we can ward off all the evil...I don't want to get diabetes. I don't want to have a stroke.”* (Male, age range 50-60 yrs.)

Second, sibling and parent morbidity and mortality from CVD was an influential context. Participants depicted this as a feeling of vulnerability about risk, and the more fatalistic sense that future CVD was inevitable for them. Third, giving and receiving care within social networks was an important illness context. Those with onerous household carer responsibilities depicted their own health as a lower priority, despite describing willingness to do more in terms of healthier lifestyle. Situations of social isolation were apparent in several female participants who responded positively to email and text message information as evidence of outside contact, and being cared about as the carer. This enhanced well-being and for some, introduced ideas for dietary and physical activity recommendations that were within their capability to try, thereby giving them more options within often-constrained routines. The weight of context in some responses to the intervention was clear:

*“They [messages] quite often come when I was a bit down. The only contact I have, usually, is with the kids. It was an outside contact, which I don't have much of. It was like somebody else cares.”* (Female, age range 60-70 yrs.)

*“just to look at the [messages] and remind me about my, how I’ve got to make sure I have my tablets and just things like this that I just felt not alone if you know what I mean, that someone was caring that I was going to be all right.”* (Female, age range 70-80 yrs.)

#### Receptiveness to risk and prevention information

Viewing personal CVD risk information (as opposed to simply having access to it) was likely interlinked with illness-related issues mentioned above. Those who felt overall well-informed about heart disease opted out of information receipt. Contexts of non-use of EHR-derived risk factor information were seen where there was seemingly illogical reasoning, distrust of risk data, or underestimation of CVD risk.

*“I just have this high cholesterol and sugar. That's all. I have no drive or interest or whatever that would urge me to go into it and find out about my condition.”* (Male, age range 60-70 yrs.)

In a similar vein, a participant with multiple risk factors whose absolute CVD risk score estimate was high but who had a normal coronary angiogram test commented:

*“I got this news that says your heart’s like a 30 year old, your blood vessels are as clear as a whistle, and I cling to that and I say well you know maybe I don’t need to do anything after all”.* (Male, age range 60-70 yrs.)

Negative emotional responses to the intervention, such as fear, were disengaging where information felt too burdensome. Other non-users disliked being reminded of what they either already knew or should be doing

*“You could be totally creamed by the amount of information… Do I need to know that? Is that going to help me to be happy, or extremely anxious? And I have had a history of panic disorder…I don’t want to go there again.”* (Female, age range 60-70 yrs.)

*“No thank you. You'd be paranoid all day.”* (Female, age range 60-70 yrs.)

Together, such responses *competed* with the program intent. In contrast, where information intersected with a context of desire to improve health it was empowering; depicted as curiosity, changed focus, increased motivation, and a sense of control - *complementing* the program intent.

*“I read most of the stuff that was on there. But once I was into the routine of it, then I was only interested in the graphs, the weight measurement, the blood pressure, And then I continually flick back to look if the heart risk had moved! [laughing]”* (Male, age range 60-70 yrs.)

*“I would like to have the remaining years as trouble free as I can…and I have been interested in, for some time, my diet and knowledge on what I should do to keep myself as well as I can for as long as I can. It's not going to do me any harm and it may do me some good”.* (Male, age range 80-90 yrs.)

#### History of the doctor-patient relationship

The history of the doctor-patient relationship and the dynamics of the health encounter were contextual and pre-dated the intervention. A perception of being well-managed and sufficiently satisfied with or reliant on information directly from the general practitioner (GP) appeared to blunt the response to the intervention resources for some, particularly the EHR-related features. Others strengthened their role in the health care relationship by using resources that improved understanding of and adherence to their treatment, and communication with their provider. Often these were participants who described feeling responsible for their own health, alongside active and long-standing engagement with their GP. This combination of circumstances influenced feeling comfortable to discuss side-effect information they had read about within the intervention’s medication screens, to query prescriptions, and to ask about alternative medication.

*“I need to know to feel relaxed and comfortable about what I’m doing. So I go and see what the side-effects are going to be…then if they do happen then I’m a little bit more aware of it and then I can watch and follow for a few days and then go back and say, ‘Well, this is happening. Could it be related?’ If something’s not pressing, I’m not concerned about anything, I don’t worry about it, but I know it’s there.”* (Female, age range 60-70 yrs.)

*“I looked on, and I thought, that’s funny, I think he’s got the medications different to what the cardiologist said. So I thought I’d better make an appointment and go and sort that out. So that was kind of really useful.”* (Female, age range 60-70 yrs.)

Agreement between provider advice and intervention content appeared to enhance response to the intervention by making it more meaningful. No participant described use of the intervention in a context of a poor relationship with his or her GP; however, several participants described themselves in terms of a “naughty” or “disobedient” child if they did not act on treatment advice or felt unsuccessful in reaching treatment goals.

#### Relationship with technology

Relationship with technology in everyday life was notable in how it explained *non-use* of this particular intervention. For example, use was minimal in several participants who described themselves as early technology adopters, and frequent, multi-application users. A tech-enabled lifestyle appeared to heighten expectations of the intervention’s functions and performance that, when unmet or when exceeded by other devices or software they used, deterred use.

*“I have an Apple phone and an iPad and an Apple watch and all that sort of thing…and they do more for me…they measure my exercise for the day, they measure the standing times, the numbers of stairs I walk up, they measure my heartbeat for me, they you know keep records of all of these things as an ongoing hour by hour.”* (Male, age range 60-70 yrs.)

Non-use was also described by participants with lower overall technology adoption, literacy and access:

*“I feel I’m part of a generation that just missed out. When I was in school, computers were the size of your kitchen fridge. And they were something we never thought about. They weren’t part of our lives, they weren’t like this…So it isn’t as driving imperative for me to be technically connected to anything.”* (Female, age range 60-70 yrs.)

However, some of these participants described using a computer for non-health activities such as games, social media and email, suggesting that eHealth literacy may be an important sub-skill. For non-computer users, mobile phone use was enabling of information receipt. As previously noted, this held important value as a proxy for care in some participants. Higher educational attainment and greater likelihood to try new technologies was noted among some participants who felt sufficiently knowledgeable about CVD prevention and sensed low benefit from the intervention. Interestingly, low educational attainment, low/no computer use and lower likelihood to try new technologies were characteristic of several participants who reported benefitting from the CVD information component; and were characteristic of those who preferred in-person health information from trusted clinic staff and did not use the intervention.

## Discussion

In this study, a realist evaluation approach was used to elucidate change mechanisms within a complex eHealth intervention and to identify and explain influential contexts on CVD prevention behaviours or intervention non-use. Technology approaches to chronic disease prevention increasingly target risk factor improvement by consumers using self-directed home-based interventions. Whilst a RCT determines overall effectiveness of such interventions, process evaluation enriches understanding of the relationship between intervention inputs and outputs by examining *how* outcomes occurred when an intervention is used in open, changeable social systems. Observable outcomes are readily identifiable. By contrast, the interaction of resources from an intervention with the recipient’s context of use (an interaction that potentially changes reasoning or decision-making about behaviour) is harder to observe but can be uncovered with use of a theory-based evaluation approach. Such concepts of realism are proposed to be plausible within a randomised trial despite its methodological intent to control confounding factors, identify aggregate intervention efficacy, and make generalisable findings [33]. Counter arguments contend that a RCT cannot identify the underlying relationships between intervention, outcome, context, and mechanism that are central to understanding causation from a realist perspective [34, 35]. The CONNECT RCT was not designed as a realist trial per se; however, examination of how the complex intervention worked (or did not work) to influence behaviour was undertaken using realist principles, for example by using the initial CONNECT program theory to inform the qualitative data analysis [36].

This study found that change mechanisms were often affective or attitudinal responses to intervention resources: for example, changed awareness, or feeling more confident, encouraged, motivated, and cared about. Noted cognitive responses included knowledge-seeking, CVD risk insight, changed reasoning about advantages of healthier behaviour, personal responsibility and capability in activating desirable lifestyle behaviours and medication adherence. Participants described important behavioural outcomes, such as adoption of, or increase in, CVD prevention recommendations and better medication adherence; also such biometric changes as lower blood pressure and weight. Cases of improved healthcare navigation and engagement were seen in terms of more active communication with care providers, medication self-management, and accountability to personal risk factor control. Intervention responsiveness ranged from apparent inertia to high engagement. Atypical responses to the intervention offered insights into contexts and conditions in which assumptions about this type of intervention may not apply. Combining the contexts of intervention use and non-use revealed four overarching social narratives were at play. Together, they provide a more general picture of conditions within an individual’s micro- and meso-level environments that could favour or limit success of future similar interventions.

### Refining the original program theory

The way the intervention worked can be explained in additional ways to that originally intended with the Fogg model and persuasive software principles. For example, descriptions of rationalising and processing risk information emerged from interviews in this study. Participants with an equivocal attitude to improving health behaviours began actively doing so in key areas of lifestyle. Some described new cognitive awareness of important relationships (such as cholesterol level and five-year CVD risk) and an advantage to making changes now to avoid future ill health. Conceptually, some participants’ responses illustrated movement within the stages of change model, [37] in that their reasoning reflected the notions of pre-contemplation, contemplation, preparation and action as a result of interaction with the intervention. Although not oriented to context, and acknowledging that stages may not progress linearly, the model’s progression construct is a useful depiction of changed awareness, commitment, and action described by participants as they engaged with the content of the intervention within their circumstances. Similarly, frameworks such as normalisation process theory describe that sense-making by individuals, or ascribing meaning, within a health related complex intervention precedes action [38]. Cognitive responses that are positive (for example, perceived relevance or benefit) or negative (for example, perceived fear or irrelevance) therefore facilitate or inhibit the incorporation of the desired actions into everyday behaviour [38]. Further, behavioural control, one’s perception of how easy or difficult it is to perform the new behaviour, [39] was another theoretical concept at play; for example, in the way that suggestions of better lifestyle choices could be applied in individual circumstances to make new habits take hold and the frequent references by interviewees to self-responsibility and empowerment. Hence, notions around increased agency emerged as an explanation of how the intervention worked for some. Central to the notion of people as agents and not just undergoers of experiences is the idea of intentionality: taking up actions for a purpose [40]. This accords with realist attribution of agency to individuals, rather than viewing them as passive recipients that an intervention ‘happens to’ [9].

In the original program theory for the intervention, viewable EHR-derived data as well as the more generic CVD prevention recommendations were assumed to provide triggers and motivation for healthier behaviour and/or prompt discussion about CVD risk factor management with healthcare providers. Some interviewees challenged this assumption. CVD information per se was not of universal value for this purpose, proving a deterrent in some circumstances but enabling in others, and in unintended ways, such as humanising an essentially automated intervention. This finding raises the broader role of context in how disease risk information is received and acted upon. Within social cognitive theory, for example, knowledge of health risks and benefits must precede behaviour change but one must perceive having control over healthful behaviour, given one’s situational facilitators and barriers [41]. Essentially, self-efficacy is linked to expectations of whether new lifestyle efforts will succeed *and* whether obstacles can be overcome. Further, health communication that raises belief in self-efficacy and places less emphasis on fear or illness vulnerability is suggested as more enabling as a means to adopting healthful behaviours [41]. Interviews with primary and secondary CVD prevention participants within the current study revealed increased health behaviour efficacy through program use, even with future disease risk as an explicit motivation. Overall, triggers, motivation and capacity as drivers of behaviour change, supplemented by persuasive software design, accounted for positive intervention effects but likely understated the role of contexts of use. It is also clear that more nuanced forces occurred in the change process between software inputs and the observable outputs of the intervention, and these may not have been explicit in the original hypotheses of how the intervention would work. Further research could formalise a revised or expanded program theory for a similar intervention for testing in more specific contexts.

### Socio-cultural narratives as influencers of intervention use

In this study, factors at the micro-level are defined as those more proximate to the individual, such as illness experience, or family and social networks [22]. Meso-level factors refer to those aspects of social structure between an individual (micro-level) and national systems (macro-level); therefore, examples of meso-level environments are workplaces, community groups, and healthcare relationships and settings [22]. The current study accords with a study of patients’ secondary CVD prevention behaviour, wherein sociocultural factors at the micro- and meso-level, such as intercurrent illnesses, social networks and healthcare system relationships, influenced prevention uptake behaviour beyond the acute hospitalization event [16]. For chronic illness self-management more generally, an individual’s community-level (non-household) social relationships appear to also confer an advantage for psychosocial and practical needs, in addition to disease self-care. In a longitudinal study of people with heart disease and diabetes, for example, these advantages extended to lower health service utilisation (and therefore costs) by those with high social network support [42]. Although such studies underscore social network as an important condition for success with disease self-care, participants in the current study frequently emphasised their personal responsibility for successful CVD prevention behaviours. Reliance on others, if it occurred, was less evident. Their descriptions of greater agency and self-efficacy resonate with policy-imperatives for individual responsibility for behaviour modification and disease risk management, [43].

Similar to the current study, a realist evaluation of a mobile application for diabetes self-management identified that disease awareness, self-efficacy and user expectations of web-based tools influenced program uptake; also, disease emotional distress was an important barrier to engagement [26]. Personalised CVD risk profile in this intervention was intended to arouse awareness of risk/vulnerability (but not fear) within the wider intent of prompting information seeking, including with the primary health provider. Yet interviewee reactions were found to be mixed along a continuum from low engagement to feeling incentivised, underscoring again that different psychosocial contexts may explain varying responses to the same resource. Interestingly, a study of illness beliefs among secondary prevention patients noted that where current symptoms were minimal, future illness risk poorly predicted uptake of preventive lifestyle behaviours [44]. Another study of determinants of CVD prevention noted higher willingness among those with lower education and in subjects with existing CVD compared with those with a family history but no personal experience [45]. On one hand, this aligns with the current study insofar as interviewees with existing coronary heart disease (but no current symptoms) referred to the significant place of that illness experience in their outlook about future health. By contrast, however, interviewees in this study with family history were engaged with risk prevention even if engagement with the actual intervention was low.

Contexts of vulnerability may be a worthwhile focus for further study of self-directed web-based prevention resources. In a setting of diabetes self-management, low socio-economic resources have shown negative influence on food purchases, motivation, knowledge, and social ties as a source of information [17]. In the current study, no participants mentioned financial barriers to healthier lifestyle behaviour choices; however, low social engagement and perceived low access to heart health information were conditions that actually favoured use of the intervention. Interestingly, previous research has noted that disease risk information for CVD [46] and diabetes [47] on freely available websites cater poorly for those with low health literacy and therefore interpretation of the information within the GP encounter is crucial. We may have under-theorised the role of participants’ relationship with their GP. Culture, for example, has been shown to account for generational differences in use of internet-mediated health care support versus virtual passivity within long-established relationships with GPs [21]. Adherence to CVD prevention medications has been identified as a behaviour embedded in the dynamics of the wider family carer roles characteristic of some cultures [20]. Interestingly, in the current study no participants mentioned family network as either a help or hindrance to treatment adherence, instead describing a strong preference for independent medication self-management in cooperation with a trusted GP. Other research has explored older patients’ role expectations within health-related decision-making as a contextual factor in itself [48]. Unsurprisingly perhaps, where the patient felt that their informal caregivers expected them to relinquish decisions to their care provider(s), the more passive they believed their role to be [48]. Other studies caution that where there are expectations of self-management as part of a health system-level agenda of greater active involvement in one’s health care processes, [43, 49] some patients cannot do so (or have no wish to do so); a context in which medical paternalism may be valued over active participation [50]. Therefore, the values by which patients actively or passively relate to their care provider – and how this changes over time – are a contextual factor (at the meso-level) in how patients with CVD or other chronic conditions engage with disease self-management. With the promise of eHealth approaches to self-care that encourage patient engagement using biometric data, for example, further research on this emerging role is needed to ensure that such interventions complement the patient-provider encounter, whatever its nature.

This research is not without limitations. First, interviewee selection was by a researcher and inadvertent bias in selectivity is a noted susceptibility of using diverse, dissimilar interviewee responses to examine the intervention’s effects. Second, evaluating the intervention through the realist approach introduced the risk of self-report bias as well as necessitated researchers making inferences about the unobservable mechanisms of intervention effect, both of which are threats to study validity [29]. Cases of intervention non-use were included to reduce this risk. Third, although generalisation of findings and wider population representativeness was not the intent, the sample size was small and the change mechanisms identified are not exhaustive. Finally, only participant experiences within the previous 12 months could be explored. Responses that occurred later may have affected behaviour beyond the time of study participation. As a result, there may be additional mechanisms that work in such an intervention when events in people’s lives change the context of use.

## Conclusions

EHR-integrated eHealth interventions have potential to aid the affective, cognitive and behavioural drivers of changing health behaviour. Four key interactive resources within such an intervention assisted patients with, or at increased of, CVD to obtain heart health outcomes that were meaningful for them. Realist evaluation as a theory-driven approach to understanding processes within a RCT uncovered the diversity of invisible change mechanisms that were activated within various contexts to produce outcomes. To optimise the supportive benefit of eHealth interventions for behaviour change, knowledge of key contextual narratives should assist in planning targeted effectiveness studies, as well as inform further research into intervention-context interactions in similar interventions.

## Supplementary information

**Additional file 1.** Application of consolidated criteria for reporting qualitative studies (COREQ) to the interview data collection.

**Additional file 2.** Contexts-mechanisms-outcomes within four interactive features of an eHealth intervention.

## Data Availability

The datasets generated and/or analysed during the current study are not publicly available due to the interview transcripts containing identifying information but are available from the corresponding author on reasonable request to the relevant ethics committees to researchers who meet the criteria for access to confidential data.

## Abbreviations

ANZCTR: Australia New Zealand Clinical Trials Registry
C-M-O: context-mechanism-outcome
CONNECT: Consumer Navigation of Electronic Cardiovascular Tools
CVD: Cardiovascular disease
eHEALS: electronic health literacy scale
EHR: Electronic health record
GP: General practitioner
RCT: Randomised controlled trial
SD: Standard deviation
