# Supplementary material for: A realist evaluation approach to explaining the role of context in the impact of a complex eHealth intervention for improving prevention of cardiovascular disease
Source: BMC Health Serv Res. 2020 Aug 18;20:764. doi: 10.1186/s12913-020-05597-5 (PMC7433103; doi:10.1186/s12913-020-05597-5)
Supplement: Supplementary file 1 — Additional file 1. Application of consolidated criteria for reporting qualitative studies (COREQ) to the interview data collection. [file 12913_2020_5597_MOESM1_ESM.pdf]

Supplementary file 1: Application of consolidated criteria for reporting qualitative studies (COREQ) to the interview data collection

Checklist Source: Tong A, Sainsbury P, Craig J. Consolidated criteria for reporting qualitative research (COREQ): a 32-item checklist for interviews and focus groups. Int J Qual Health Care. 2007;19(6):349-357

No.	Item	Guide questions/description
**Domain 1: Research team and reflexivity**		
Personal Characteristics		
1.	Interviewer/facilitator	Which author/s conducted the interview or focus group?<br>GC
2.	Credentials	What were the researcher's credentials? *E.g. PhD, MD*<br>BAppSc, MA, MPH
3.	Occupation	What was their occupation at the time of the study?<br>Senior Project Officer
4.	Gender	Was the researcher male or female?<br>Female
5.	Experience and training	What experience or training did the researcher have?<br>Training from senior study academic; previous experience of focus group conduct.
Relationship with participants		
6.	Relationship established	Was a relationship established prior to study commencement?<br>Telephone contact with some interviewees had occurred during the routine conduct of the RCT
7.	Participant knowledge of the interviewer	What did the participants know about the researcher? e*.g. personal goals, reasons for doing the research*<br>Reasons for doing the research were provided to each interviewee in an information sheet; discussed again when the interview was requested and again before written consent was obtained.
8.	Interviewer characteristics	What characteristics were reported about the interviewer/facilitator? e.g. *Bias, assumptions, reasons and interests in the research topic*<br>Potential for selection bias was acknowledged as a study limitation within the manuscript.

No.	Item	Guide questions/description
**Domain 2: study design**		
Theoretical framework		
9.	Methodological orientation and Theory	What methodological orientation was stated to underpin the study? *e.g. grounded theory, discourse analysis, ethnography, phenomenology, content analysis*<br>The study was informed by Realist Evaluation theory and results for the contextual narratives were thematically derived from the interview data.
Participant selection		
10.	Sampling	How were participants selected? *e.g. purposive, convenience, consecutive, snowball*<br>Purposive.
11.	Method of approach	How were participants approached? e.*g. face-to-face, telephone, mail, email*<br>Telephone
12.	Sample size	How many participants were in the study?<br>36 of the participants from the intervention arm of the RCT (n=486) took part in an interview.
13.	Non-participation	How many people refused to participate or dropped out? Reasons?<br>Seventeen participants who were approached for an interview declined to take part for reasons such as disinterest, illness, or hospitalisation.
Setting		
14.	Setting of data collection	Where was the data collected? e.*g. home, clinic, workplace*<br>Primary health care services.
15.	Presence of non-participants	Was anyone else present besides the participants and researchers?<br>No
16.	Description of sample	What are the important characteristics of the sample? *e.g. demographic data, date*<br>Use/non-use of the intervention; demographic data; presence of diagnosed cardiovascular disease (CVD) or presence of CVD risk factors without diagnosed CVD
Data collection		
17.	Interview guide	Were questions, prompts, guides provided by the authors? Was it pilot tested?<br>An ethically approved semi-structured interview guide was used by the researcher but not provided to the interviewees or pilot tested.

No.	Item	Guide questions/description
18.	Repeat interviews	Were repeat interviews carried out? If yes, how many?<br>There were no repeat interviews.
19.	Audio/visual recording	Did the research use audio or visual recording to collect the data?<br>Audio recording of the interviews was used.
20.	Field notes	Were field notes made during and/or after the interview or focus group?<br>Field notes were made after the interviews for personal journaling of the researcher’s reflections on the interview process; however, the contents were not used in the analysis of results.
21.	Duration	What was the duration of the interviews or focus group?<br>Interviews lasted up to one hour.
22.	Data saturation	Was data saturation discussed?<br>Yes, by two researchers (GC and JR).
23.	Transcripts returned	Were transcripts returned to participants for comment and/or correction?<br>The transcribed audio recordings of the interviews were not returned to participants to proofread. For several logistical reasons, doing so was not part of the study protocol, nor part of the participant informed consent; therefore, participants were not expecting to receive them and their participation was not conditional on them proofing the interview transcript.
**Domain 3: analysis and findings**		
Data analysis		
24.	Number of data coders	How many data coders coded the data?<br>One
25.	Description of the coding tree	Did authors provide a description of the coding tree?<br>No
26.	Derivation of themes	Were themes identified in advance or derived from the data?<br>Derived from the data
27.	Software	What software, if applicable, was used to manage the data?<br>NVivo 12 Pro
28.	Participant checking	Did participants provide feedback on the findings?<br>No
Reporting		

No.	Item	Guide questions/description
29.	Quotations presented	Were participant quotations presented to illustrate the themes / findings? Was each quotation identified? e.*g. participant number*<br>Yes, quotes were presented, identified by sex and age of the interviewee.
30.	Data and findings consistent	Was there consistency between the data presented and the findings?<br>Yes
31.	Clarity of major themes	Were major themes clearly presented in the findings?<br>Yes
32.	Clarity of minor themes	Is there a description of diverse cases or discussion of minor themes?<br>Yes, what we called ‘negative cases’ were included and described to ensure that the results incorporated how the identified themes both facilitated or impeded intervention uptake.
