# Supplementary material for: A realist evaluation approach to explaining the role of context in the impact of a complex eHealth intervention for improving prevention of cardiovascular disease
Source: BMC Health Serv Res. 2020 Aug 18;20:764. doi: 10.1186/s12913-020-05597-5 (PMC7433103; doi:10.1186/s12913-020-05597-5)
Supplement: Supplementary file 2 — Additional file 2. Contexts-mechanisms-outcomes within four interactive features of an eHealth intervention. [file 12913_2020_5597_MOESM2_ESM.pdf]

Supplementary file 2: Contexts-mechanisms-outcomes within four interactive features of an eHealth intervention

1. EHR-derived risk score estimation; biometric and pathology risk profile (receptiveness to personalised CVD risk profile information)	
**Examples of responses to the intervention feature, or reasoning expressed**	*"It sort of woke me up. Well the cholesterol and the heart risk, I never even used to think about until I was doing this."* (Male, age range 60-70 yrs.)<br><br>*"Well, you know statistically where you stand. Like, what's the likelihood or possibility of having some heart situation, incident, within a certain number of years… but then also how that can be reduced…and I thought, wow, that's pretty motivating in itself."* (Male, age range 70-80 yrs.)<br><br>*"I was thinking everything I was doing alright but then you sort of realise, well, with not a whole lot of effort, if you're just a bit more conscious of it, you can do better."* (Male, age range 70-80 yrs.)
**Sub-themes present in contexts of use (or non-use)**	▪ feeling vulnerable to CVD because siblings and/or parents have CVD<br>▪ fear of poor future health from observing parents' health in decline<br>▪ unexpected intensification of drug therapy to control CVD risk factors
**Change mechanisms**	▪ raised consciousness about relationship of modifiable CVD risk factors with behaviour<br>▪ motivated towards healthier lifestyle behaviour;<br>▪ moved from being unconcerned or unaware to concrete engagement with personal CVD risk profile
**Outcomes reported**	▪ increased physical activity<br>▪ healthier eating habits and food choices

2. **CVD guideline-recommended lifestyle information and medication tips.**<br>**(Receptiveness to information aimed at 'nudging' the recipient towards healthier lifestyle behaviour/choices)**	
**Examples of responses to the intervention feature, or reasoning expressed**	*"It just got me thinking. Like I should be doing something."* (Female, age range 40-50 yrs.)<br><br>*"…planting that seed in the back of my head that yes, I've got to do that. It nudges you to something, I don't know, you tend to go and do it. So it's not necessarily the message, it's just, for me to say, okay, I need to think about myself."* (Female, age range 50-60 yrs.)<br><br>*"I think it's important to be told what to eat…and you know just things that you shouldn't eat. I just felt so sure of being looked after and, and knowing what to do."* (Female, age range 70-80 yrs.)
**Sub-themes present in contexts of use (or non-use)**	▪ feeling socially isolated<br>▪ feeling that own health takes lower priority in a household of high carer demands<br>▪ wishing to improve health and make lifestyle changes because of a concerning blood test result; or concern about future health due to multiple current risk factors/concurrent illnesses
**Change mechanisms**	▪ felt supported and cared about<br>▪ raised health consciousness<br>▪ felt motivated, confident and incentivised to action<br>▪ changed perception about healthier lifestyle choices being feasible within current routine<br>▪ anticipation of benefit
**Outcomes reported**	▪ eating to dietary recommendations<br>▪ increased weekly physical activity<br>▪ weight loss<br>▪ improved blood pressure control<br>▪ increased feelings of well-being<br>▪ more assertive about asking questions during medical appointments

3. Updateable medication list from the EHR, with consumer drug information (Prescription medication knowledge)	
**Examples of responses to the intervention feature, or reasoning expressed**	*"I like to know what I'm putting in my body and how it interacts with other medicines and things like that…I don't suffer side effects very much but every now and then something will creep in that's not usual, and I'll be able to go back and have a look. So I can deal with it. I don't have to sort of ring [GP] up every five minutes or be on his doorstep every five minutes."* (Female, age range 60-70 yrs.)<br><br>*"I need to know to feel relaxed and comfortable about what I'm doing. So I go and see what the side-effects are going to be…then if they do happen then I'm a little bit more aware of it and then I can watch and follow for a few days and then go back and say, 'Well, this is happening. Could it be related?' If something's not pressing, I'm not concerned about anything, I don't worry about it, but I know it's there."* (Female, age range 60-70 yrs.)
**Sub-themes present in contexts of use (or non-use)**	▪ feeling responsible for one's health, including knowing about prescriptions and effects<br>▪ active and long-standing engagement with the general practitioner
**Change mechanisms**	▪ reassurance derived from understanding medications facilitated adherence and heightened feelings of self-care agency
**Outcomes reported**	▪ improved medication adherence<br>▪ increased dialogue with prescriber(s) and pharmacist to ensure accuracy and understanding of prescriptions<br>▪ increased confidence with managing the medication regimen

**4. Personalised goal setting and tracking with virtual rewards (Tracking lifestyle health behaviour goals)**	
**Examples of responses to the intervention feature, or reasoning expressed**	*"I can't work... and that's why getting involved in the study was helpful because…the activity section of the goal-setting thing was "Oh well, I'd better go and make sure I cross off that this week and get that done". You know, I used to plan it in with my so-called weekly new normal."*<br>(Male, age range 50-60 yrs.)<br><br>*"It is good to stimulate you and give you purpose…so that you think, well I achieved that today, I felt good about that."* (Female, age range 60-70 yrs.)<br><br>*"to see the things that have a positive impact on you, to see it there in front of you on the screen and realise that instead of having whatever it might be three times a week, it would be just as easy to have five times a week."* (Male, age range 70-80 yrs.)<br><br>*"When it comes to this New Year's resolutions type stuff, never been into doing that."*<br>(Male, age range 60-70 yrs.)<br><br>*"[Logging in and tracking goals] is something you've got to go and do…I couldn't be bothered."*<br>(Male, age range 60-70 yrs.)
**Sub-themes present in contexts of use (or non-use)**	▪ having a goals orientation in working life and other pursuits; interest in measurable activities<br>▪ feeling at risk for CVD and having thought about lifestyle changes but not enacted them<br>▪ experiencing illness that curtailed employment and previous activities; structured activity planning was an antidote to isolation and boredom<br>▪ wanting a way to prioritise dietary and physical activity guidelines into weekly routine<br>▪ general disinterest in formal goal tracking;

	▪ feeling disciplined enough in health-related behaviour that electronic resources are unnecessary<br>▪ prefers to use face-to-face health resources<br>▪ interested in goal setting for healthier lifestyle but unenthusiastic about computer use
**Change mechanisms**	▪ raised health consciousness<br>▪ desirable behaviours became more habitual<br>▪ felt incentivised to action<br>▪ positive feedback offered motivation to intensify effort<br>▪ increased agency/self-esteem in health behaviour
**Outcomes reported**	▪ weight loss<br>▪ smoking cessation<br>▪ improved blood pressure or cholesterol control<br>▪ heathier food choices<br>▪ increased physical activity
